# Altered Amygdala Excitation and CB1 Receptor Modulation of Aggressive Behavior in the Neuroligin-3^R451C^ Mouse Model of Autism

**DOI:** 10.3389/fncel.2018.00234

**Published:** 2018-08-03

**Authors:** Suzanne Hosie, Daniel T. Malone, Stephanie Liu, Michelle Glass, Paul Anthony Adlard, Anthony John Hannan, Elisa L. Hill-Yardin

**Affiliations:** ^1^School of Health and Biomedical Sciences, RMIT University, Bundoora, VIC, Australia; ^2^Monash Institute of Pharmaceutical Sciences, Monash University, Parkville, VIC, Australia; ^3^Department of Pharmacology, University of Auckland, Auckland, New Zealand; ^4^Florey Institute of Neuroscience and Mental Health, University of Melbourne, Parkville, VIC, Australia; ^5^Department of Anatomy and Neuroscience, University of Melbourne, Parkville, VIC, Australia; ^6^Department of Physiology, University of Melbourne, Parkville, VIC, Australia

**Keywords:** autism spectrum disorder, neuroligin, synaptic currents, aggression, cannabinoid receptor, WIN-55212-2

## Abstract

Understanding neuronal mechanisms underlying aggression in patients with autism spectrum disorder (ASD) could lead to better treatments and prognosis. The Neuroligin-3 (NL3)^R451C^ mouse model of ASD has a heightened aggressive phenotype, however the biological mechanisms underlying this behavior are unknown. It is well established that NL3^R451C^ mice have imbalanced excitatory and inhibitory synaptic activity in the hippocampus and somatosensory cortex. The amygdala plays a role in modulating aggressive behavior, however potential changes in synaptic activity in this region have not previously been assessed in this model. We investigated whether aggressive behavior is robustly present in mice expressing the R451C mutation, following back-crossing onto a congenic background strain. Endocannabinoids influence social interaction and aggressive behavior, therefore we also studied the effects of cannabinoid receptor 1 (CB1) agonist on NL3^R451C^ mice. We report that NL3^R451C^ mice have increased amplitude of miniature excitatory postsynaptic currents (EPSCs) with a concomitant decrease in the amplitude of inhibitory postsynaptic currents (IPSCs) in the basolateral amygdala. Importantly, we demonstrated that NL3^R451C^ mice bred on a C57Bl/6 background strain exhibit an aggressive phenotype. Following non-sedating doses (0.3 and 1.0 mg/kg) of the CB1 receptor agonist WIN55,212-2 (WIN), we observed a significant reduction in aggressive behavior in NL3^R451C^ mice. These findings demonstrate altered synaptic activity in the basolateral amygdala and suggest that the NL3^R451C^ mouse model is a useful preclinical tool to understand the role of CB1 receptor function in aggressive behavior.

## Introduction

Autism spectrum disorder (ASD) is a highly prevalent neurodevelopmental disorder diagnosed by impaired social interaction, repetitive behavior and/or restrictive interests. In addition, multiple comorbid traits are commonly observed including hyperactivity, increased anxiety and aggressive behavior (Argyropoulos et al., 2013; Burrows et al., 2015; Farmer et al., 2015). Aggressive behavior is commonly reported for individuals diagnosed with ASD (Kanne and Mazurek, 2011; Fitzpatrick et al., 2016), with a recent study citing that 39.5% of all people with ASD demonstrated aggression, self-injurious behavior or severe tantrums (Adler et al., 2015). Importantly, a significant number of patients with ASD are drug refractory for aggressive behaviors (Farmer et al., 2015). Aggressive behavior in ASD limits patient access to education, healthcare and employment and is one of the strongest predictors for admission to institutions (Lakin et al., 1983) and poor quality of life among individuals with developmental disabilities (Gardner and Moffatt, 1990). Although the neurobiological mechanisms underlying aggression are not fully characterized, changes in neural activity within the amygdala and connectivity between the amygdala and other brain regions may contribute to these behaviors (Varkevisser et al., 2017; Buades-Rotger et al., 2018).

A large proportion of genetic mutations associated with ASD alter synaptic function (Betancur et al., 2009; Betancur, 2011; Krumm et al., 2014; Bourgeron, 2015). Multiple rodent models expressing synaptic mutations found in patients show autism-relevant traits (Tabuchi et al., 2007; Silverman et al., 2010; Peñagarikano and Geschwind, 2012; Schmeisser et al., 2012). Of particular interest is the neurexin-neuroligin-shank interactive pathway of synaptic proteins (Bourgeron, 2015). Neuroligins are expressed post-synaptically throughout the central nervous system and together with neurexins, mediate trans-synaptic signaling (Südhof, 2008; Arons et al., 2012; Peñagarikano and Geschwind, 2012; Földy et al., 2013). Disruption of neuroligins and neurexins impairs synaptic function without completely abolishing synaptic transmission (Chubykin et al., 2005). Here, we utilized a gene-edited mouse model to study the role of the synaptic adhesion protein Neuroligin-3 (NL3). A mutation in NL3 has been identified in patients diagnosed with ASD, one of which exhibited ongoing and problematic aggressive behaviors (Jamain et al., 2003). NL3^R451C^ mice express a missense mutation causing an arginine residue to be replaced by a cysteine at position 451, resulting in a drastic reduction in the amount of protein expressed at the membrane (Chubykin et al., 2005; Südhof, 2008).

Mice expressing the R451C mutation in NL3^R451C^ bred on a mixed background strain exhibit increased aggressive behavior, which was rescued following administration of the atypical antipsychotic risperidone (Burrows et al., 2015), however the underlying biological mechanisms responsible are unknown. Previous studies in this model have shown changes in the endocannabinoid system in the hippocampus and cortex (Földy et al., 2013; Speed et al., 2015). On both a mixed background strain as well as mice bred on a pure C57Bl/6 background strain these mice have increased cortical inhibition (Tabuchi et al., 2007; Etherton et al., 2011; Pizzarelli and Cherubini, 2013) and enhanced hippocampal excitation in brain slices (Tabuchi et al., 2007; Etherton et al., 2011). Changes in synaptic activity in areas associated with aggression such as the basolateral amygdala, could contribute to the observed aggressive phenotype. Whether synaptic activity in the basolateral amygdala is altered in NL3^R451C^ mice has not been investigated.

A role for endocannabinoids in altered synaptic function in neurodevelopmental disorders including autism has been proposed (Busquets-Garcia et al., 2013; Prager et al., 2016). The cannabinoid receptor 1 (CB1) plays an important role in social interaction and aggressive behavior (Rodriguez-Arias et al., 2013). NL3^R451C^ mice bred on a mixed background show changes in tonic cannabinoid signaling in specific hippocampal cell types and circuits (Földy et al., 2013).

In order to examine synaptic transmission in a brain region implicated in aggressive behaviors, we assessed whether the inhibitory/excitatory balance is altered in the basolateral amygdala in the NL3^R451C^ mice. *Nlgn3* is expressed in the adult mouse amygdala (Lein et al., 2007). Given the modulatory effects of CB1 receptor activation on aggressive behavior and synaptic function, another aim of this study was to determine whether CB1 receptor activation could ameliorate aggressive behaviors in this mouse strain. This research is important in driving the identification of potential targets for improved therapeutic treatments in ASD.

## Materials and Methods

### Animal Husbandry

B6;129-Nlgn3tm1Sud/J mice were obtained from Jackson Laboratories (Bar Harbor, ME, USA) and crossed onto a C57Bl/6 background for over 10 generations (F10). NL3^R451C^ and wild-type (WT) animals were derived by mating heterozygous females with NL3^R451C^ males, which produced 50:50 WT and NL3^R451C^ offspring. Only male (Y/+ and Y/R451C) mice were used for this study. All electrophysiology experimental protocols were approved by the Florey Institute of Neuroscience and Mental Health Animal Ethics Committee and the animal behavior and autoradiography experimental protocols were approved by the Monash Institute of Pharmaceutical Sciences (MIPS) Animal Ethics Committee.

### Electrophysiology

Following anesthesia with 1%–3% isoflurane (inhalation), postnatal day 15–46 mice were decapitated and cortical slices were cut (300 μm thick). Using whole cell patch clamp technique in voltage clamp mode either miniature inhibitory or excitatory postsynaptic currents (mIPSCs/mEPSCs) were recorded from pyramidal-like neurons (selected based on their large soma size and morphology as visualized via infrared differential interference contrast optics) of the basolateral amygdala.

Brain slices were bathed in artificial cerebrospinal fluid containing 125 mM NaCl, 25 mM NaHCO_3_, 3 mM KCl, 1.25 mM NaH_2_PO_4_H_2_O, 1 mM MgCl_2_, 2 mM CaCl_2_, 10 mM glucose, aerated with 95% O_2_/5% CO_2_ to a final pH of 7.4. TTX (0.5 mM) was present in all recordings and 50 μM D-AP5 (Tocris), 10 μM 6-Cyano-7-nitroquinoxaline-2,3-dione (CNQX; Tocris) and 50 μM Picrotoxin (Tocris) were added for inhibitory and excitatory recordings respectively. Internal pipette solutions were 120 mM CsCl, 10 mM Hepes, 5 mM NaCl, 1 mM MgCl_2_, 0.3 mM NaGTP, 3 mM MgATP, 10 mM EGTA, 5 mM QX-314, 8 mM biocytin hydrochloride for inhibitory, and 117.5 mM CsMeSO_4_, 10 mM Hepes, 10 mM tetra-ethylammonium chloride (TEA-Cl), 15.5 mM CsCl, 1 mM MgCl_2_, 10 mM Na phosphocreatine, 8 mM NaCl, 0.3 mM NaGTP, 4 mM MgATP, 5 mM EGTA, 1 mM QX-314, 8 mM biocytin hydrochloride for excitatory recordings.

For each cell, synaptic events were detected using the template matching algorithm of pClamp 9.0 (Molecular Devices) for 3 × 3 min recordings held at a membrane potential of −70 mV and at a constant temperature of 32°C. Data were only included in analysis if the series resistance was <25 MΩ and did not change by >20% during the course of the recordings. Using analysis software (Clampfit, Molecular Devices, San Jose, CA, USA) the peak amplitude and inter-event interval of these post synaptic currents were measured and compared. The results were plotted as a cumulative fraction curve allowing the average population of events over a 3 min recording to be shown. Data was also compared using mean values over the entire 3 min recording period and plotted as a box plot.

In order to confirm the approximate location of each cell recorded an image of the patch pipette and whole-cell was acquired using a Sony (CCD XC-ST50CE) camera attached to an Olympus BX-51 microscope. These images were overlaid onto a plate image from the Mouse Brain Atlas (Paxinos and Franklin, 2001) corresponding to figure 42 (Bregma −1.34 mm, interaural 2.046 mm) using the imaging software Corel Draw Graphic Suite X7.

### Animal Behavior

Mice were weaned from their mother at postnatal day 21 and allowed to habituate to a 12 h dark/light cycle (lights on at 2200, lights off at 1000) in the holding room for at least 7 days before initiation of juvenile social interaction testing at postnatal day 28 ± 3. Locomotor activity testing was conducted at postnatal day 56 ± 5, and resident intruder testing was conducted at postnatal day 73 ± 7.

### Juvenile Social Interaction

At postnatal day 28, each mouse was isolated in a clean cage in a separate room 1 h prior to testing. Mice were paired with littermates or non-littermates based on weight and placed in opposite corners of a 39.5 × 39.5 cm perspex arena lined with fresh sawdust bedding. Interactions between WT-WT, NL3-WT and NL3-NL3 pairs were assessed by counting the number of incidences of the following behaviors: anogenital sniffing, head sniffing, self-grooming (grooming for a minimum of 2 s), jumping and rearing and were defined as per Chadman et al. (2008).

Each mouse was tested for juvenile social interaction once, with the exception of one mouse that was paired with two different mice for separate interaction recordings on different days. Behavior was recorded for 10 min, as reported previously (Chadman et al., 2008). Between each test, sawdust bedding in the arena was disposed of and replaced with fresh bedding. Recordings of mouse behaviors were assessed manually by a single observer blinded to genotype and treatment.

### Resident-Intruder Test

Using the well-established Resident-Intruder assay (Miczek et al., 2001; Koolhaas et al., 2013), territorial aggression behaviors were assessed by monitoring aggressive behavior displayed by the resident (subject mouse) when a novel juvenile mouse was placed into the resident’s home cage for a period of 5 min.

At postnatal day 66 ± 7 resident mice (i.e., WT or NL3) were habituated in single cages. C57Bl/6 WT intruder mice were obtained from Monash Animal Services (Monash University, Clayton) on the same day as resident mice to allow 7 days of habituation into the dark/light cycle of the holding room before testing. To differentiate intruder mice from residents, a small patch of fur on their upper back was shaved using animal clippers.

At postnatal day 73 ± 7, following 7 days of social isolation, resident mice were weight-matched with a younger intruder mouse at an age of postnatal day 45–56. In order to minimize influences of variable weight between juvenile intruders and resident mice, weights were taken into consideration when allocating pairs. Weight matching was achieved with a mean weight difference of 2 g. Resident mice were paired with the same intruder mice for four consecutive days. Resident mice were injected with either vehicle (20% captisol^®^, a polyanionic cyclodextrin derivative in water) or the CB1 receptor agonist WIN55,212-2 at either 0.3 or 1 mg/kg 20 min before testing. The intruder mouse was then placed into the home cage of the resident and interactions were recorded for 5 min by video camera. Neither the genotype nor the drugs administered were known to the observer at the time of video analysis. Parameters scored were: attack latency (latency to first attack), attack incidence, attack duration, and the number of occurrences of anogenital sniffing, head sniffing, mounting and number of tail rattles. An attack was recorded when one mouse approached the other mouse at high speed to bite (usually on the back) and grasped the mouse using forepaws, sometimes rolling over multiple times in order to maintain contact while biting.

### Statistical Analysis

Cumulative curves describing the distribution of miniature synaptic events (amplitude and inter-event interval) were compared using the non-parametric Kolmogorov-Smirnov statistical test (GraphPad Prism software). Mean values for amplitude and inter-event interval for miniature synaptic events for each genotype were also compared using a Student’s *t*-test. One-way ANOVA was used to analyze juvenile social interaction data and the Holm-Sidak’s multiple comparison test was used to differentiate significance between groups. Locomotor activity assessment (LMA) and aggression test parameters were analyzed using two-way ANOVA. When a main effect was detected, a Tukey’s multiple comparisons test was conducted to evaluate significant differences.

## Results

### Altered Excitability in Basolateral Amygdala

**Figure 1:**
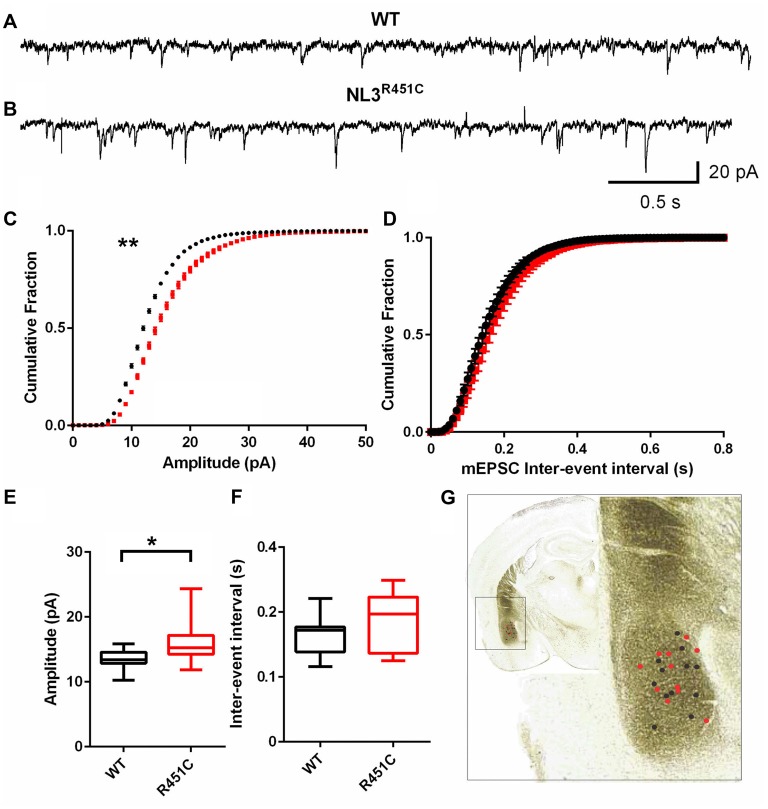
Increased miniature excitatory postsynaptic current (mEPSC) amplitude in neuroligin-3 (NL3) ^R451C^ mice. **(A,B)** Example traces of mEPSCs recorded from pyramidal-like neurons in the basolateral amygdala. **(C,D)** Cumulative fraction analysis of the mean population of mEPSC amplitude and inter-event interval respectively. **(E,F)** Mean amplitude and inter-event interval of mEPSCs. Wild-type (WT) *n* = 12 cells recorded from seven animals and NL3 *n* = 11 cells recorded from seven animals. **(G)** Approximate location of recorded cells overlaid on an image of a coronal brain section. *P < 0.05, **P < 0.01; ***P < 0.001.

Synaptic currents were recorded from pyramidal-like neurons of the amygdala in the NL3^R451C^ and WT littermate controls. The age of the animal was not found to impact the post synaptic recordings (data not shown) so data was pooled. Raw traces of mEPSCs from WT and NL3^R451C^ mice demonstrated an increase in event amplitude in mutant mice (Figures 1A,B). A comparison of cumulative fraction amplitude curves showed larger amplitudes for NL3^R451C^ mice than WT littermates (Figure 1C; *P* = 0.0096). No differences in the inter-event interval in NL3^R451C^ and WT mice were observed (Figures 1D,F). In agreement with the analysis of cumulative fraction curves, the mean amplitude of events in NL3^R451C^ mice was larger (Figure 1E; *P* = 0.016). The location of each recorded cell is shown overlaid on an image of a coronal section from the Mouse Brain Atlas (Paxinos and Franklin, 2001; Figure 1G).

**Figure 2:**
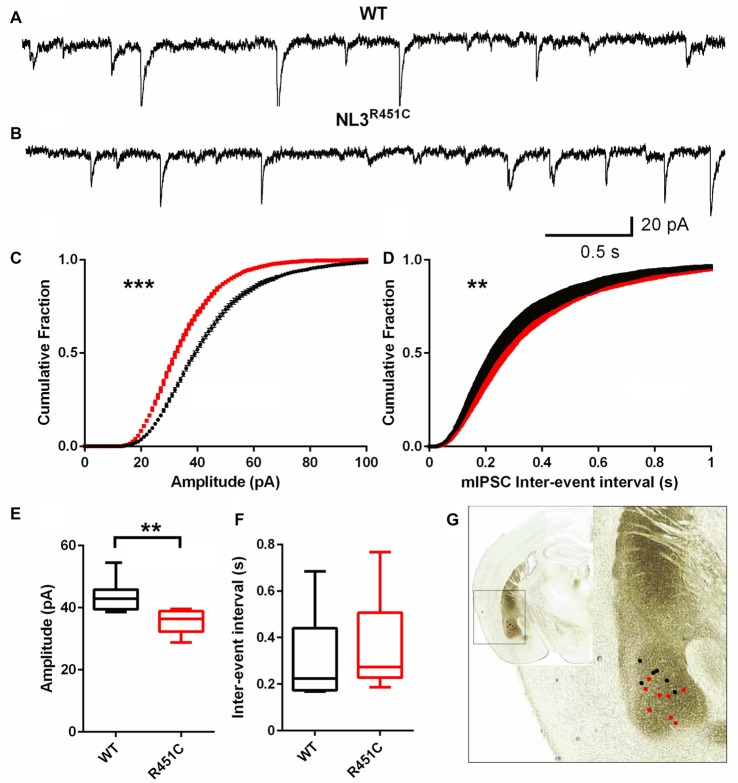
Decreased miniature inhibitory postsynaptic current (mIPSC) amplitude and increased frequency of events in NL3 ^R451C^ mice. **(A,B)** Example traces of mIPSC recorded from basolateral amygdala pyramidal-like neurons. **(C,D)** Cumulative fraction analysis of mean population of mIPSC amplitude and inter-event interval respectively. **(E,F)** Mean amplitude and inter-event interval of mIPSCs. WT *n* = 7 cells recorded from four animals NL3 *n* = 8 cells recorded from three animals. **(G)** Approximate location of mIPSC recording on image of coronal brain section *P < 0.05, **P < 0.01; ***P < 0.001.

Inhibitory currents had reduced amplitude and an increased inter-event interval in NL3^R451C^ mice (Figure 2) as shown by representative raw traces (Figures 2A,B) and cumulative fraction curves (Figures 2C,D). The mIPSC peak amplitude in neurons from NL3^R451C^ mice was significantly smaller than in WTs (Figure 2C; *P* = 0.0008). When analyzed using the Kolmogorov Smirnov test, inter-event interval was increased in NL3^R451C^ mice (Figure 2D; *P* = 0.001). The mean event amplitude was significantly smaller in NL3^R451C^ compared to WT littermates (Figure 2E; *P* = 0.005). When comparing mean values, the inter-event interval was unchanged between genotypes (Figure 2F). The location of each recorded cell is shown in Figure 2G (inset).

### Increased Frequency of Jumping in Juvenile Mixed-Genotype Pairs

To determine if behavioral phenotypes including aggression caused by the R451C mutation penetrate across background strains we examined a range of behavioral outputs in WT and NL3^R451C^ mice bred on a pure C57Bl/6 background strain. Freely interacting juvenile mice (4 weeks of age) were observed for frequency of self-grooming, head-sniffing and ano-genital sniffing, jumping and rearing.

**Figure 3:**
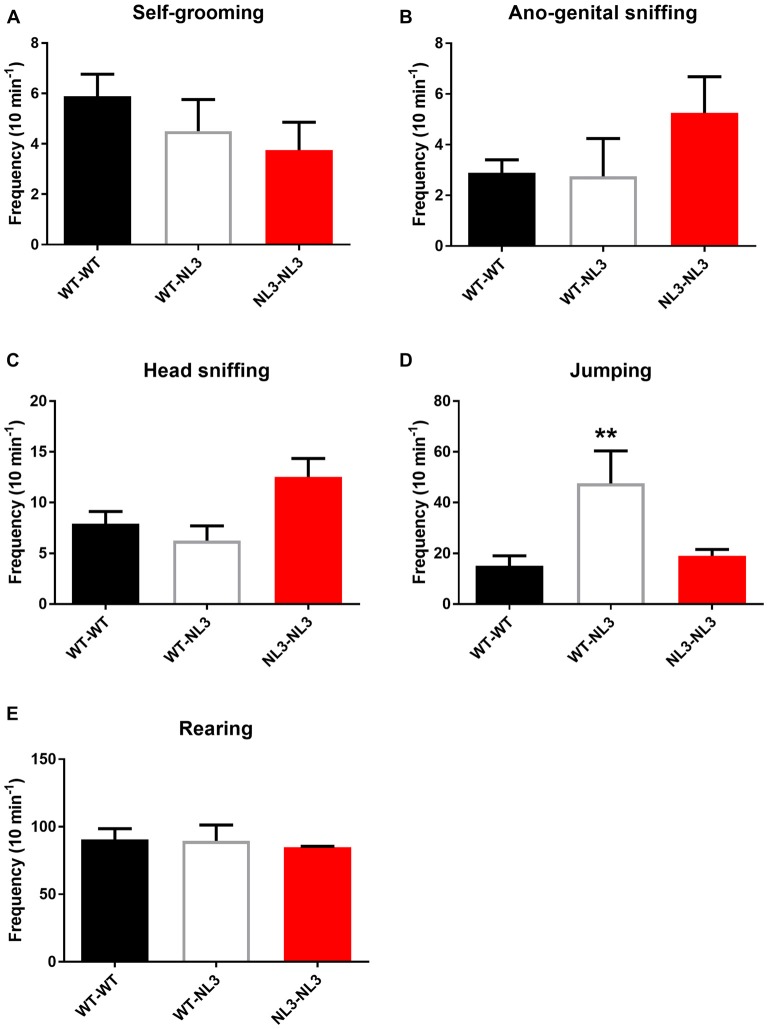
Juvenile social interaction is altered in mixed genotype pairs. **(A–E)** Juvenile social interaction behavioral parameters analyzed between pairs of mice following recordings of interactions during 10 min. Results are expressed as mean and error bars represent SEM WT-WT *n* = 9 pairs (*n* = 18 mice), WT-NL3 *n* = 4 pairs (*n* = 8 mice), NL3-NL3 *n* = 4 pairs (*n* = 8 mice) **P < 0.01.

There were no significant differences observed between the WT/WT pairs and NL3^R451C^/NL3^R451C^ pairs for self-grooming, head-sniffing, ano-genital sniffing and rearing (Figures 3A–C,E). There was a main effect of genotype on jumping behavior (*F*_(2,14)_ = 6.669; *P* = 0.0092; Figure 3D). Mixed genotype pairs (WT/NL3^R451C^) showed an increase in jumping events compared to the WT/WT (*p* = 0.009) and NL3/NL3 (*P* = 0.0356) pairs as revealed by the Holm-Sidak’s multiple comparison test.

### Abnormal Aggression in NL3^R451C^ Mice

**Figure 4:**
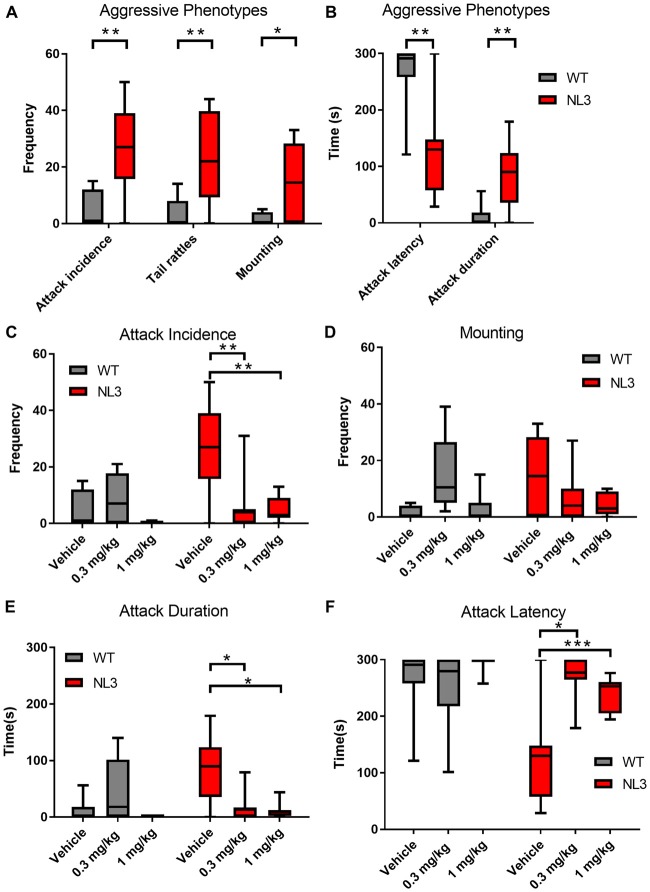
Aggressive behaviors in NL3 mice are modified by the non-selective CB receptor agonist WIN55,212-2. **(A,B)** Comparison of aggressive parameters (mounting, tail rattles, attack incidence, attack duration attack latency over 5 min) between NL3 ^R451C^ and wild type littermates. **(C–F)** The effect of WIN55,212-2 (0.3 mg/kg and 1.0 mg/kg) on aggressive parameters. Results were averaged over four consecutive days and expressed as mean ± SEM. (WT *n* = 8, NL3 *n* = 8, WT/WIN55,212-2 0.1 mg/kg *n* = 7, NL3/WIN55,212-2 0.1 mg/kg *n* = 7, WT/WIN55,212-2 0.3 mg/kg *n* = 8, NL3/WIN55,212-2 0.3 mg/kg *n* = 7, *P < 0.05, **P < 0.01, ***P < 0.001).

To investigate the possibility of NL3^R451C^ behavior being regulated through a CB1 receptor pathway, we tested for aggressive behaviors in mice following administration of the CB1 receptor agonist WIN 55, 212–2 (0.3 and 1 mg/kg). Aggressive behavioral parameters including the number of mounting episodes and tail rattles displayed as well as attack incidence, attack duration and attack latency were compared between NL3^R451C^ mice and WT littermates using the resident-intruder test (Figure 4). Overall, we identified a significant effect of genotype on all parameters reported and a drug effect for attack incidence, duration and latency (ANOVA). NL3^R451C^ mice exhibited more mounting behavior and tail rattles (Figure 4A) compared to WT littermate controls (*P* = 0.0439 and *P* = 0.0172 respectively). The incidence (Figure 4A) and duration of attacks (Figure 4B) were also greater in the NL3^R451C^ mice (*P* = 0.0002 and *P* = 0.025 respectively). Furthermore, attack latency was reduced in mutant mice (*P* = 0.0001) compared to WT littermates (Figure 4B). These findings suggest increased aggressive behaviors in NL3^R451C^ mice compared to wild-type littermates.

Administration of WIN55,212-2 (0.3 and 1 mg/kg) caused no change in the occurrence of mounting behavior or tail rattles in either the NL3^R451C^ or WT littermates (*F*_(2,19)_ = 1.953; *P* = 0.1693 and *F*_(2,19)_ = 1.103; *P* = 0.3521 respectively; ANOVA repeated measures; Figure 4D and data not shown). WIN55,212-2 altered attack incidence (*F*_(2,19)_ = 8.214; *P* = 0.0027; duration *F*_(2,19)_ = 7.73; *P* = 0.0035) and latency of attack (*F*_(2,19)_ = 9.79; *P* = 0.0012; ANOVA repeated measures). *Post hoc* analyses (Tukey) identified that in NL3^R451C^ mice, administration of WIN 55, 212–2 reduced attack incidences (*P* = 0.0096 for 0.3 and *P* = 0.0051 for 1 mg/kg), attack duration (*P* = 0.0125 for 0.3 and *P* = 0.0063 for 1 mg/kg), and increased attack latency (0.3 mg/kg *P* = 0.0063 and 1 mg/kg *P* = 0.0026; Figures 4C,E,F).

In order to clarify the potential sedative effects of WIN55,212-2 at 0.3 and 1 mg/kg, the locomotor activity was recorded and observed. There was no effect of genotype on either the track length (*F*_(1,36)_ = 0.062; *P* = 0.805) or average velocity (*F*_(1,36)_ = 0.0041; *P* = 0.949) measured. Similarly, there was no effect of drug on track length (*F*_(1,36)_ = 2.653; *P* = 0.084) or average velocity (*F*_(1,36)_ = 2.067; *P* = 0.14 respectively; Supplementary Figures S1A,B).

To further understand the behavioral differences, CB1 and GABA_A_ receptor radioligand binding was examined using autoradiography. Radioligand binding at these receptors was found to be normal in the prefrontal cortex (*P* = 0.7634), hippocampus (*P* = 0.6020), VMH (*P* = 0.9147), amygdala (*P* = 0.3860) and PAG (*P* = 0.3860; Supplementary Figures S2A,B) compared to the WT littermates. There were also no significant differences found in the density of GABA_A_ receptors in the prefrontal cortex (*P* = 0.4114), hippocampus (*P* = 0.0766), VMH (*P* = 0.1725), amygdala (*P* = 0.3362) and PAG (*P* = 0.6425; Supplementary Figures S2C,D). This suggests that the NL3 gene mutation had no effect on expression of these receptors and that the behavioral effects of the CB1 receptor agonist (WIN55,212-2; Figure 4) were not secondary to altered receptor levels.

## Discussion

It is well established that NL3^R451C^ mice display autism-relevant behaviors (Chadman et al., 2008; Etherton et al., 2011; Rothwell et al., 2014; Tabuchi et al., 2007). In the current study, mixed-genotype juvenile WT/NL3 pairs showed increased jumping compared with WT/WT and NL3/NL3 pairs, which could be an indication of hyperactivity as has been demonstrated in another mouse model of autism (Shank2 KO mice; Schmeisser et al., 2012), although this only occurred in mixed genotype pairs. Interestingly, recent advances in automated analyses of behavioral data have enabled accurate tracking of mouse group dynamics (Chaumont et al., 2018). In assessing two genetic mouse models of ASD, Shank2 and Shank3 KO mice, this approach also identified atypical social behavior that disrupted group interactions when mixed genotypes were present but not for groups of mice with the same genotype. With greater uptake of such technology, underlying differences in mixed genotype behavioral changes compared to genotype-matched mouse groups may be better understood in the context of the NL3^R451C^ model.

We have previously shown that NL3^R451C^ mice demonstrate a robust aggressive phenotype when bred on a mixed-background strain (Burrows et al., 2015). Here, we show that the aggressive phenotype persists on a congenic C57Bl/6 background strain as seen by increased incidence and duration of attacks. Attack latency was also reduced. In addition to these attack related measures, we also observed increased mounting behavior and frequency of tail rattles in mice bred on a pure C57Bl/6 background strain expressing the R451C mutation further confirming a role for NL3 in aggressive behavior. This study therefore provides clear evidence that the aggressive phenotype is not due to susceptibility genes inherent in the background strain or solely environmental effects as the phenotype is also present on the pure C57Bl/6 strain. Further, the aggressive phenotype shown in the C57Bl/6 mice is more easily compared across laboratories than a mixed strain background which would show quite different genetic drift with breeding over time.

The NL3 R451C mutation alters cannabinoid signaling in specific neuronal subtypes and thus impacts the cannabinoid neuronal regulation pathway (Földy et al., 2013; Speed et al., 2015). To determine whether cannabinoid modulators affect aggressive behavioral parameters in NL3^R451C^ mice, we investigated changes in aggression interactions in the presence of the CB1 receptor agonist WIN 55, 212–2. Overall, administration of WIN 55, 212–2 at 0.3 and 1 mg/kg rescued the aggressive phenotype in NL3^R451C^ mice. CB1 receptors are highly expressed in the adult mouse amygdala (Mackie, 2005; Marsicano and Lutz, 1999), further supporting the potential involvement of this brain region in regulating heightened aggression in NL3^R451C^ mice. Although the CB1 receptor is broadly expressed in multiple brain regions, its role is modulating the neural circuitry relevant to aggression in NL3^R451C^ mice is unknown. The integrated activity of brain regions generates complex behaviors such as aggression, therefore in addition to changes in the basolateral amygdala it is likely that other brain regions will also be altered by the R451C mutation. The involvement of cannabinoids in modulation of aggressive behavior in NL3^R451C^ mice is in line with previous reports showing that CB1 agonists strongly decrease aggressive behavior as well as findings that CB1 KO mice are more aggressive than WT control mice (Rodriguez-Arias et al., 2013).

In order to obtain further understanding of the mechanisms underlying the behavioral phenotype in these mice, we examined neuronal activity in the amygdala, a brain region associated with aggression. Our data demonstrates altered synaptic activity in a brain region that has not previously been investigated in these mice. Specifically, we have demonstrated decreased inhibitory postsynaptic activity alongside an increase in excitatory post-synaptic activity onto pyramidal cells in the basolateral amygdala. Previous reports of abnormal synaptic activity in these mice strongly suggest that alterations in activity are cell-type and synapse-type dependent (Földy et al., 2013). For example, Földy et al. (2013) showed specific changes in synaptic activity in CCK-immunoreactive neurons synapsing with pyramidal neurons in the hippocampus and that these alterations are mediated by tonic inhibition due to changes in endocannabinoid neurotransmission. In the current study, we show increased excitation and a reduction in GABAergic synaptic events in the basolateral amygdala, a brain region implicated in aggressive behaviors (Varkevisser et al., 2017; Buades-Rotger et al., 2018). We propose that this alteration in neural activity could contribute to the aggressive phenotype we have observed in these mice. Cannabinoid receptors are expressed broadly on excitatory and inhibitory neurons in the rodent brain (Kawamura et al., 2006; Hill et al., 2007). The finding that aggressive behavior is reduced to control levels in NL3^R451C^ mice following administration of WIN-55, 212–2 suggests that cannabinoid agonists may alleviate abnormal synaptic activity and assist in identifying new approaches to improve challenging behaviors in the clinic when synaptic mutations are involved. These novel findings further confirm the face validity of the R451C mutation and demonstrate that the aggressive phenotype persists in mice bred on the C57Bl/6 background strain.

A previous report showed increased levels of the vesicular GABA transporter (vGAT) using immunolabelling and quantitative immunoblotting in NL3^R451C^ mice bred on a mixed background strain (Tabuchi et al., 2007) suggesting a potential mechanism for increased inhibitory neurotransmission via GABA_A_ receptors in the somatosensory cortex. In contrast, when we assessed for potential changes in GABA_A_ receptor density using autoradiography we did not identify any genotype specific alterations. This may indicate that potential changes in receptor density are below the detection threshold of the autoradiography technique, which gives an overall indication of expression in specific brain regions.

In summary, this study shows that the NL3 R451C mutation confers a heightened aggression phenotype in C57Bl/6 mice that is reversed by administration of the CB1 agonist, WIN-55, 212–2 and that mice in mixed genotype pairs show increased jumping; potentially indicating hyperactivity. We further demonstrate synaptic dysfunction in the basolateral amygdala, an area that may contribute to the aggressive behavioral phenotype in this model. Further studies to determine the precise effects of these synaptic changes as well as potential rescue effects of WIN-55, 212–2 on synaptic function in multiple brain regions in these mice are required in order to inform the design of novel targeted clinical therapies.

## Author Contributions

EH-Y, AJH and DM: designed research study. SH, MG and SL: conducted experiments. SH, DM, AJH, PAA and EH-Y: writing manuscript.

## Conflict of Interest Statement

The authors declare that the research was conducted in the absence of any commercial or financial relationships that could be construed as a potential conflict of interest.
