# Supplementary material for: Altered Amygdala Excitation and CB1 Receptor Modulation of Aggressive Behavior in the Neuroligin-3R451C Mouse Model of Autism
Source: Front Cell Neurosci. 2018 Aug 3;12:234. doi: 10.3389/fncel.2018.00234 (PMC6085410; doi:10.3389/fncel.2018.00234)
Supplement: Supplementary file 1 [file Data_Sheet_1.pdf]

SUPPLEMENTARY MATERIAL

Supplementary Information

Locomotor activity

At postnatal day 56 ± 5, mice were individually acclimatised to a 39.5 x 39.5 cm perspex arena (Alternative Plastics, North Melbourne, Australia) for 10 minutes. On the consecutive day, vehicle or WIN55,212-2 (0.1mg/kg or 0.3mg/kg) was administered to each test mouse in a separate room in dim light (lux level of 2.1). The mouse was then isolated for 20 minutes before locomotor activity (LMA) testing was conducted in the perspex arena for 10 minutes. Between each test, the arena was cleaned with ethanol and allowed to dry. Distance travelled was measured by analysing all video footage using the tracking software ViewerIII (BiObserve, Germany). Activity was analysed during 10 minutes of recording footage as well as activity in each sector of a 3 x 3 grid of the arena to exclude unexpected bias in certain sectors. In order to determine an overall measure of LMA, both tracklength and velocity were measured.

Receptor autoradiography

6 NL3 mice and 6 WT mice (P56-107) were habituated in a 12 hour dark/light cycle holding room for 3 days to minimise potential anxiety due to relocation before culling. The brain was removed from each mouse and placed on dry ice within 2 minutes of culling, then stored in the -80°C freezer for 4 days.

Autoradiography was performed as previously reported (Glass, et al., 1993) at the Centre for Brain Research, University of Auckland, New Zealand. Fresh frozen brain tissue was cut coronally on a Leica CM1950 cryostat at a thickness of 16 micron. Sections were mounted on double dipped gelatin slides and stored at -80°C prior to radiolabelling. Slide-mounted cryostat sections were removed from the -80°C freezer and rapidly air-dried immediately preceding radiolabelling.

Multiple brain regions were selected for analysis based on previous reports implicating their importance in mediating aggressive behaviours: prefrontal cortex (van Erp & Miczek, 2000), hippocampus (Becker et al, 1999; Nehrenberg et al., 2009), ventromedial hypothalamus (VMH) (Lin et al., 2011; Nehrenberg et al., 2009) amygdala (Nehrenberg et al., 2009) and the periaqueductal grey (PAG) ((Bhatt & Siegel, 2006; Nehrenberg et al., 2009). Tissue slices were obtained from the prefrontal cortex (~Bregma 2.58), hippocampus (also used for visualisation of receptor binding in the amygdala and ventromedial hypothalamus) (~Bregma -2.06) and periaqueductal gray (~Bregma -2.70). These regions were defined according to Paxinos and Franklin (2004).

For $GABA_A$ receptor labeling, tissue was incubated in 1nM [^{3}H]flunitrazepam (Amersham, GE Healthcare) in 50 mM Tris-HCl (pH 7.4) at 4°C for 60 minutes before two 1 minute washes in ice cold 50 mM Tris-HCl (pH 7.4). Slides were then rinsed with ice-cold distilled water. Non-specific binding was determined by incubation with 1 µM flumazenil (Tocris, Bristol, UK) as the displacer.

CB_1 receptors were labeled with 2.5nM [^{3}H]CP55,940 (DuPont/NEN, Boston, MA, USA) in 50 mM Tris-HCl with 5% bovine serum albumin and incubated at 37°C for 120 minutes. Slides were then washed twice for 2 hours each with Tris-HCl with 1% bovine serum albumin. Non-specific binding of CB1 receptors was determined by 10 µM CP55,940 (Tocris, Bristol, UK) as the displacer (Glass et al., 1993). After radiolabeling, all slides were fan-dried overnight at 4°C. Slides were exposed to tritium sensitive Kodak Biomax MR film (Carestream Health Inc.,

Rochester, NY, USA) for 6 weeks and 10 weeks for $GABA_A$ and CB_1 receptors respectively. Films were developed (Kodak D19 developer at 4 °C; 7 min), water rinsed (30 s), fixed (Ilford Hypam rapid paper and film fixer; 6 min), washed in water (5 min) and dried prior to high resolution scanning. Mean grey levels were measured from the specified regions: prefrontal cortex, hippocampus, VMH, amygdala and PAG using ImageJ software (NIH, USA). The receptor binding values presented were calculated as the difference between total binding (binding in the presence of the radioligand alone) and non-specific binding (binding in the presence of the radioligand and a saturating concentration of a displacing ligand). Non-specific binding was measured in an adjacent section from the same anatomical region.

Unpaired t-tests were used to compare receptor expression between WT and NL3 mice. A significance level of $P<0.05$ was used.

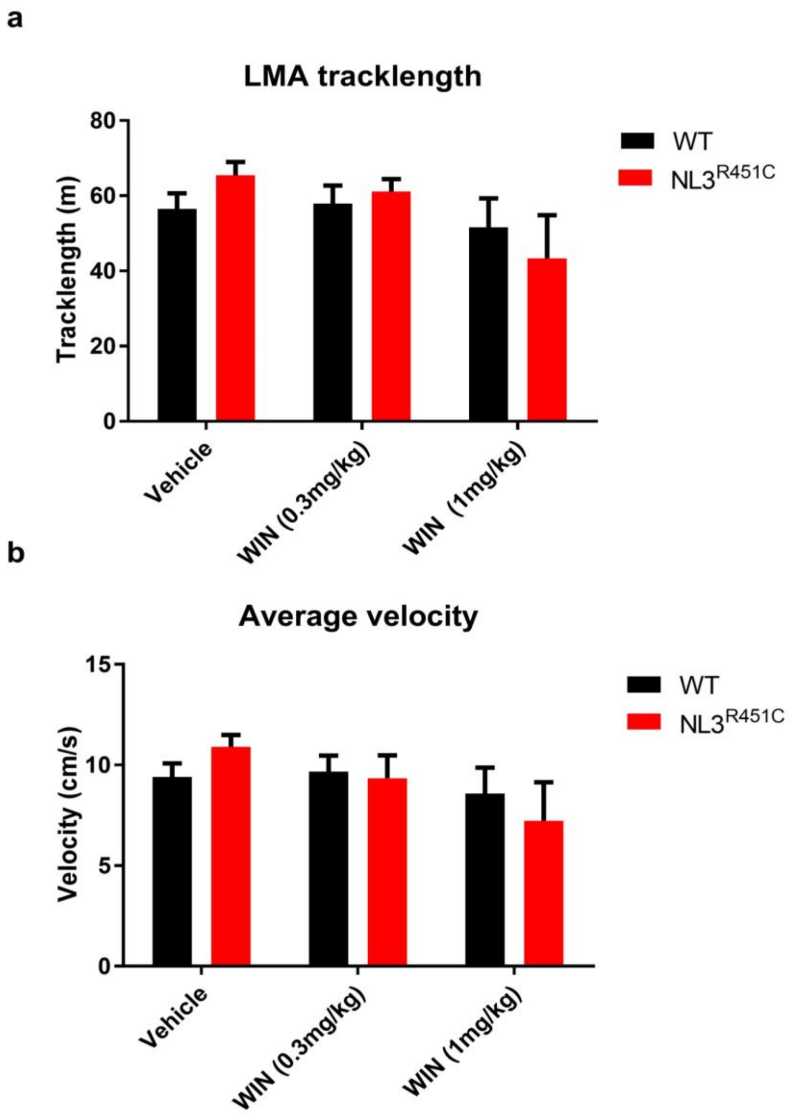

S 1 WIN-55,212-2 has no impact on locomotor activity

a, Effect of WIN-55,212-2 on LMA tracklength and **b**, velocity (WT/Vehicle n=8, NL3/Vehicle n=8, WT/WIN-55,212-2 0.3mg/kg n=6, NL3/WIN-55,212-2 0.3mg/kg n=7, NL3/WIN-55,212-2 1mg/kg n=6, NL3/WIN-55,212-2 1mg/kg n=7). Results expressed as mean and SEM.

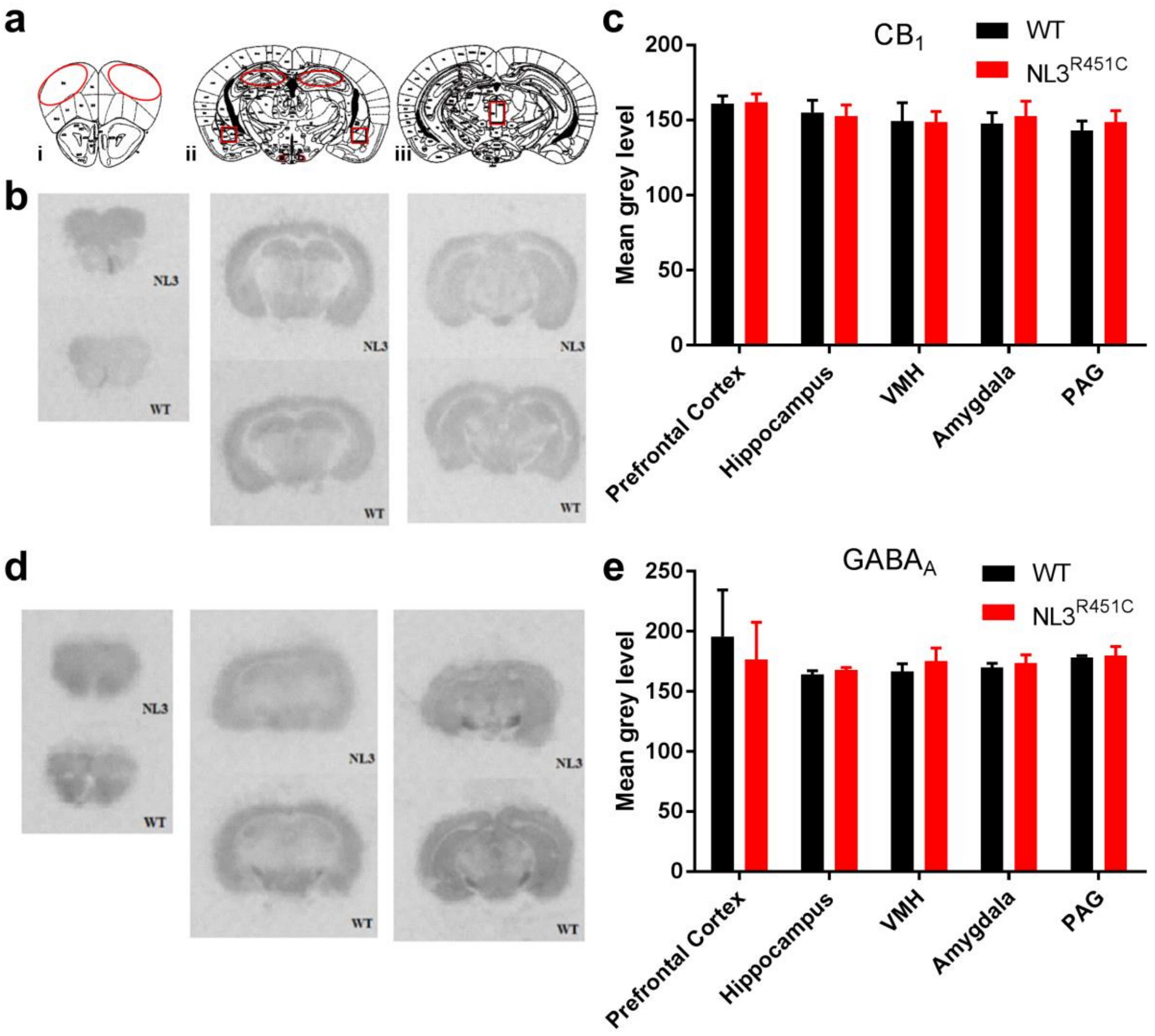

S2 Autoradiography for expression of CB1 and GABA$_A$ receptors.

a, Location of measurement of mean grey levels for i, prefrontal cortex (bregma 2.58), ii, hippocampus (red oval, mean grey level taken as an average of left and right sides, bregma -2.06), amygdala (large red square, mean grey level taken as an average of left and right sides), ventromedial hypothalamus (small red square, mean grey level taken as an average left and right sides) and iii, PAG (red rectangle, bregma -2.70). **b, d,** Images of mouse brain sections showing CB1 and GABA$_A$ receptor expression respectively. **c, e,** Analysis of autoradiograms for CB$_1$ receptor and GABA$_A$ expression between NL3^{R451C} and WT mice.

References

Becker, A., Grecksch, G., Bernstein, H. G., Hollt, V., & Bogerts, B. (1999). Social behaviour in rats lesioned with ibotenic acid in the hippocampus: quantitative and qualitative analysis. *Psychopharmacology (Berl), 144*(4), 333-338.

Bhatt, S., & Siegel, A. (2006). Potentiating role of interleukin 2 (IL-2) receptors in the midbrain periaqueductal gray (PAG) upon defensive rage behavior in the cat: role of neurokinin NK(1) receptors. *Behav Brain Res, 167*(2), 251-260. doi: 10.1016/j.bbr.2005.09.011

Glass, M., Faull, R. L., & Dragunow, M. (1993). Loss of cannabinoid receptors in the substantia nigra in Huntington's disease. *Neuroscience*, 56(3), 523-527.

Lin, D., Boyle, M. P., Dollar, P., Lee, H., Lein, E. S., Perona, P., & Anderson, D. J. (2011). Functional identification of an aggression locus in the mouse hypothalamus. *Nature, 470*(7333), 221-226. doi: 10.1038/nature09736

Nehrenberg, D. L., Rodriguiz, R. M., Cyr, M., Zhang, X., Lauder, J. M., Gariepy, J. L., & Wetsel, W. C. (2009). An anxiety-like phenotype in mice selectively bred for aggression. *Behav Brain Res*, *201*(1), 179-191. doi: 10.1016/j.bbr.2009.02.010

van Erp, A. M., & Miczek, K. A. (2000). Aggressive behavior, increased accumbal dopamine, and decreased cortical serotonin in rats. *J Neurosci, 20*(24), 9320-9325.
